# A type II toxin–antitoxin system is responsible for the cell death at low temperature in *Pseudomonas syringae* Lz4W lacking RNase R

**DOI:** 10.1016/j.jbc.2024.107600

**Published:** 2024-07-25

**Authors:** Pragya Mittal, Anurag K. Sinha, Apuratha Pandiyan, Leela Kumari, Malay K. Ray, Theetha L. Pavankumar

**Affiliations:** 1 Centre for Cellular and Molecular Biology (CCMB), Council of Scientific and Industrial Research (CSIR), Hyderabad, India; 2 Celtic Renewables Ltd, Edinburgh Napier University, Edinburgh, UK; 3 National Food Institute, Technical University of Denmark, Kongens Lyngby, Denmark; 4 Department of Biological Sciences, Indian Institute of Science Education and Research (IISER) Mohali, Punjab, India; 5 Department of Microbiology and Molecular Genetics, University of California, Davis, California, USA; 6 Department of Molecular and Cellular Biology, University of California, Davis, California, USA

**Keywords:** cold sensitivity, cell death, cold-induced DNA damage, RNA degradation, RNA unwinding, toxin–antitoxin system

## Abstract

RNase R (encoded by the *rnr* gene) is a highly processive 3′ → 5′ exoribonuclease essential for the growth of the psychrotrophic bacterium *Pseudomonas syringae* Lz4W at low temperature. The cell death of a *rnr* deletion mutant at low temperature has been previously attributed to processing defects in 16S rRNA, defective ribosomal assembly, and inefficient protein synthesis. We recently showed that RNase R is required to protect *P. syringae* Lz4W from DNA damage and oxidative stress, independent of its exoribonuclease activity. Here, we show that the processing defect in 16S rRNA does not cause cell death of the *rnr* mutant of *P. syringae* at low temperature. Our results demonstrate that the *rnr* mutant of *P. syringae* Lz4W, complemented with a RNase R deficient in exoribonuclease function (RNase R^D284A^), is defective in 16S rRNA processing but can grow at 4 °C. This suggested that the processing defect in ribosomal RNAs is not a cause of the cold sensitivity of the *rnr* mutant. We further show that the *rnr* mutant accumulates copies of the indigenous plasmid pLz4W that bears a type II toxin–antitoxin (TA) system (*P. syringae* antitoxin–*P. syringae* toxin). This phenotype was rescued by overexpressing antitoxin psA in the *rnr* mutant, suggesting that activation of the type II TA system leads to cold sensitivity of the *rnr* mutant of *P. syringae* Lz4W. Here, we report a previously unknown functional relationship between the cold sensitivity of the *rnr* mutant and a type II TA system in *P. syringae* Lz4W.

The *Pseudomonas syringae* Lz4W is an Antarctic isolate, capable of growing between 0 and 25 °C, with an optimal growth rate at 22 °C (1). Psychrophilic microorganisms have acquired various adaptation strategies to combat the low temperature-induced stresses (2). Adaption of *P. syringae* Lz4W to low temperature has been studied at multiple cellular levels, such as plasma membranes (3, 4, 5, 6, 7), transcription (8, 9), gene regulation (10, 11), cold-active enzymes, and cold shock proteins (12, 13, 14). Further, the relevance of DNA metabolism and RNA degradation processes have also been investigated in the cold adaptation of psychrophiles and psychrotrophs (2).

DNA and RNA can both act as cellular thermometers (15). Nucleic acids undergo structural changes in response to low temperatures that can affect the growth of bacteria (16). For example, low-temperature–induced double-strand breaks during DNA replication adversely affect cellular growth and require efficient DNA repair proteins for growth at low temperature (17, 18, 19). Similarly, RNA secondary and tertiary structures stabilize at low temperatures, affecting the transcription and translation processes in the cells (20). RNA helicases and exoribonucleases stimulate the degradation of these stabilized RNAs at low temperatures through their RNA unwinding and degradation activities (16).

RNase R (encoded by the *rnr* gene) is a RNB/RNR superfamily exoribonuclease and is important for RNA processing and quality control in bacteria (21, 22, 23, 24). RNase R is a highly processive, hydrolytic, 3′ – 5′ exoribonuclease capable of degrading highly structured RNAs (25, 26). It is implicated in the degradation of repetitive extragenic palindromic sequences of mRNA, maturation of tmRNA, and processing of 16S and 5S ribosomal RNA (23, 26). In *P. syringae*, the *rnr* mutant is sensitive to oxidative stress and DNA-damaging agents (27, 28). Importantly, RNase R of *P. syringae* (RNase R^Ps^) is essential for growth at low temperature, and the *rnr* mutant is cold sensitive (23). In *P. syringae* Lz4W, RNase R associates with the RNA degradosome complex and is implicated in the maturation of 16S and 5S rRNA (25). Previously, cold sensitivity and the cell death of *rnr* mutant at low temperature was hypothesized to be due to processing defects of 16S and 5S rRNA, defective ribosomal assembly, and inefficient protein synthesis (23).

Here, we directly examined this hypothesis by studying the exoribonuclease defective mutant of RNase R and found that the exoribonuclease function of RNase R is not essential for the survival of *P. syringae* at low temperature. We further discovered that the *rnr* mutant accumulated copies of the indigenous plasmid of *P. syringae* Lz4W (pLz4W) that bears a type II toxin–antitoxin (TA) system of the RelE/ParE family. The accumulation of pLz4W was resolved by overexpressing antitoxin in the *rnr* mutant, suggesting an imbalance in the levels of toxin and antitoxin leads to cold sensitivity of the *rnr* mutant of *P. syringae* Lz4W. This study links the RNase R functionally to a type II TA system.

## Results

### The exoribonuclease function of RNase R is dispensable for the survival of *P. syringae* at low temperature

**Figure 1:**
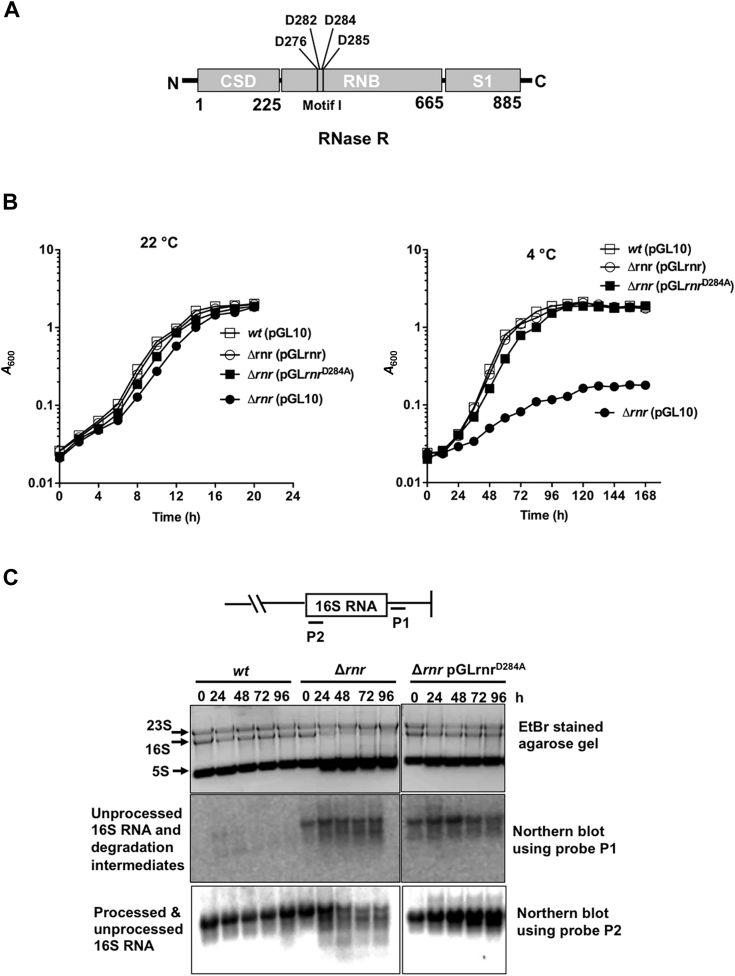
**The exoribonuclease-defective RNase R**^**D284A**^**mutant grows similar to wildtype at both 22 °C and 4 °C.***A*, a schematic representation of RNase R^Ps^. Protein domains of RNase R and the catalytically important aspartic acid residues in the morif-1 of the RNB domain are shown. *B*, growth profiles of *wt*, the Δ*rnr* stain expressing the RNase R^D284A^ mutant (pGLrnr^D284A^) at 22 °C and 4 °C. Growth of each strain was determined by measuring the absorbance of cells at 600 nm (*A*_600_) at regular time intervals as indicated. *C*, Northern analysis of 16S rRNA from the *Δrnr* strain complemented with RNase R^D284A^. *Top panel*, a schematic representation of 16S rRNA showing the positions of probe P1, specific to the region of unprocessed 3′-end of the16S rRNA and the probe P2, specific to the matured region of 16S rRNA. 15 μg of total RNA was loaded on to a 1% w/v agarose gel and electrophoresed. Northern hybridization showing the accumulated unprocessed 3′-end of 16S rRNA in both *Δrnr* and *Δrnr* complemented with RNase R^D284A^ strains upon shifting cells to 4 °C for different time points. The ethidium bromide (EtBr)-stained agarose gels used for the Northern hybridization are also shown. CSD, cold-shock domain; RNase R^Ps^, RNase R of *P. syringae*.

RNase R^Ps^ has catalytically important four conserved aspartate residues at the motif-1 of the RNB domain (Fig. 1*A*). Substitution of aspartate residue at the 284th position of RNase R with alanine (RNase R^D284A^) abolishes the exoribonuclease activity of RNase R (27). We examined the growth of wildtype (*wt*) with empty plasmid and *Δrnr* cells harboring either empty plasmid or plasmid expressing wildtype RNase R or exoribonuclease defective RNase R^D284A^ allele at 22 and 4 °C (Fig. 1*B*). We confirmed that the *rnr* mutant is cold sensitive and unable to grow at 4 °C (Fig. 1*B*, right panel). Surprisingly, the expression of RNase R^D284A^ protein suppressed the cold sensitivity of *Δrnr* cells similar to wildtype RNase R (Fig. 1*B*, right panel), indicating that the exoribonuclease function of RNase R is not required for the growth of *P. syringae* at low temperature.

The *rnr* mutant of *P. syringae* is defective in the 3′-end processing of 16S and 5S rRNAs at low temperature and the unprocessed 16S rRNA accumulates in polysomes, affecting the protein synthesis at low temperature (23). Hence, we investigated the ability of RNase R^D284A^ mutant to process 16S rRNA at low temperature. As expected, the RNase R^D284A^ mutant failed to process the 16S rRNA and accumulated the unprocessed 16S rRNA at low temperature similar to *Δrnr* cells (Fig. 1*C*). These results suggest that the defects in the processing of ribosomal RNA are unlikely to be the reason for the cold sensitivity of the *rnr* mutant. However, a function of RNase R other than its exoribonuclease activity is apparently required for the survival of *P. syringae* at low temperature.

### RNase R deleted *P. syringae* cells accumulate various forms of the native pLz4W plasmid at low temperature

**Figure 2:**
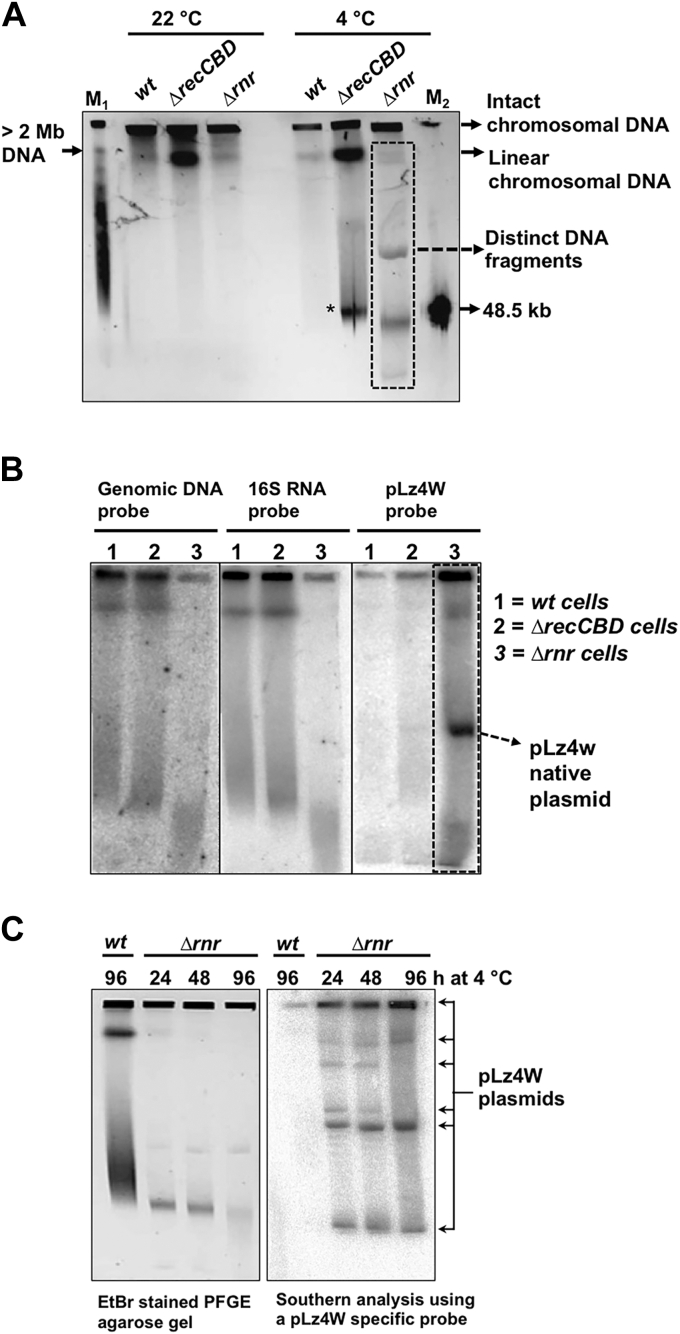
**Pulsed-field gel electrophoresis (PFGE) and Southern analysis of cellular DNAs of wild-type and mutant strains.***A*, PFGE of cellular DNAs of *wt*, Δ*recCBD and* Δ*rnr* cells grown at 22 °C and 4 °C. M1 and M2 are the yeast chromosomal and low-range PFGE markers from New England Biolabs (USA). Ethidium bromide (EtBr)-stained gel showing intact (circular) chromosomal DNA in the well, linearized chromosomal DNA of >2 mb in size, and the broken DNA fragments of sizes around 50 kb are also indicated. Note that the discrete DNA fragments of various sizes are observed only in Δ*rnr* cells incubated at 4 °C. *B*, Southern analysis of PFGE resolved cellular DNAs of *wt*, Δ*recCBD, and* Δ*rnr* cells at 4 °C. Southern hybridization using probes specific to the chromosomal DNA, 16S RNA, and the indigenous plasmid pLz4W. A probe specific to pLz4W hybridizes to the discrete DNA fragments of Δ*rnr* cells incubated at 4 °C. *C*, time-dependent accumulation of discrete DNA fragments in Δ*rnr* cells at 4 °C and the Southern analysis using a probe specific to pLz4W. *Δrnr* cells show a time-dependent accumulation of various forms of pLz4W at 4 °C. pLz4W, plasmid of *P. syringae* Lz4W.

Recently, we showed that RNase R protects *P. syringae* cells from oxidative stress and DNA damage (27). Hence, we hypothesized that RNase R may either directly or indirectly be involved in DNA damage and chromosomal fragmentation at low temperature. To understand the extent of DNA damage and chromosomal fragmentation in *Δrnr* cells at low temperature, we subjected *wt* and *Δrnr* cells to pulsed-field gel electrophoresis (PFGE) as described in the Experimental procedures. The resolution of chromosomal DNA of *wt* and *Δrnr* cells grown at 22 °C showed no apparent difference in their resolved DNA fragments (Fig. 2*A*). Intriguingly, the PFGE analysis of genomic DNA of *Δrnr* cells grown at 4 °C showed the accumulation of several distinct larger DNA fragments (Fig. 2*A*, highlighted box). Note that RecBCD, an enzyme involved in homologous recombination and DNA repair, deleted *P. syringae* cells (*ΔrecCBD*), used as a control, exhibited accumulation of large amounts of linearized and broken chromosomal DNA fragments (of >2 mb and ∼50 kb in sizes) at low temperature as observed earlier (Fig. 2*A*) (17, 19).

To identify the source of distinct DNA fragments observed in *Δrnr* cells at 4 °C, we further subjected the PFGE agarose gels to Southern hybridization using the αP^32^-radiolabeled probes specific to either chromosomal DNA or ribosomal RNA or to an indigenous pLz4W plasmid DNA. The distinct DNA fragments observed in *Δrnr* cells at 4 °C and much of the DNA in wells, while growing at 4 °C, were highlighted using the pLz4W plasmid as a probe suggesting the accumulation of various forms of pLz4W plasmid (Fig. 2*B*). To further corroborate this finding, we incubated *Δrnr* cells at 4 °C for the time intervals as indicated and analyzed the cellular DNA by PFGE and Southern hybridization using a specific probe to pLz4W (Fig. 2*C*). *Δrnr* cells accumulate increasing amounts of DNA fragments upon incubation for the different time intervals at 4 °C. These fragments clearly hybridized to the probe specific to pLz4W, suggesting that they originated from the pLz4W plasmid (Fig. 2*C*).

On conventional agarose gels, plasmid DNA runs with various mobilities depending on its supercoiled (form I), open/nicked/relaxed circular (form II), and linear (form III) forms (29). Under pulsed-filed gel electrophoresis conditions, the mobilities of various forms of plasmid are reportedly anomalous and produce various DNA bands of relatively high molecular size (30, 31). We have also observed such anomalous mobility of plasmids in pulsed-field gel electrophoresis as reported earlier (32).

To further understand the nature of mobility and accumulation of native pLz4W plasmid in general, we examined the mobility patterns and the levels of a control plasmid, pGL10 (a broad-host vector of 8.5 kb in size with IncP replicon), in *wt* and *Δrnr* cells. In this experiment, we grown *wt* and *Δrnr* cells harboring pGL10 plasmid at 4 °C and analyzed them by PFGE (Fig. S1, left panel) and Southern hybridization using a radio-labeled probe specific to pGL10 (Fig. S1, right panel). To verify the mobility of a plasmid under PFGE conditions, we ran the purified pGL10 plasmid (lanes labeled as pGL10). As shown in the Fig. S1, the pGL10 plasmid runs at various levels with altered mobilities suggesting that the different forms of plasmid DNA run anomalously as reported earlier (30, 31, 32). It is also noticeable that some plasmid DNA remained trapped in the wells. The DNA trapped in the wells is likely to be the nicked/relaxed-circular form of pGL10 (Fig. S1, right panel) as reported earlier (30, 31, 32). These different forms of plasmids possibly originate during the processing of DNA samples for PFGE. Importantly, these data show that unlike pLz4W, the levels of pGL10 remain unchanged in both *wt* and *Δrnr* cells at 4 °C (Fig. 2*C* *versus* Fig. S1, right panel), indicating the increase in levels of DNA is specific to the pLz4W plasmid.

### The *E. coli* RNase R and the exoribonuclease-defective RNase R^Ps^ both alleviate the levels of pLz4W

**Figure 3:**
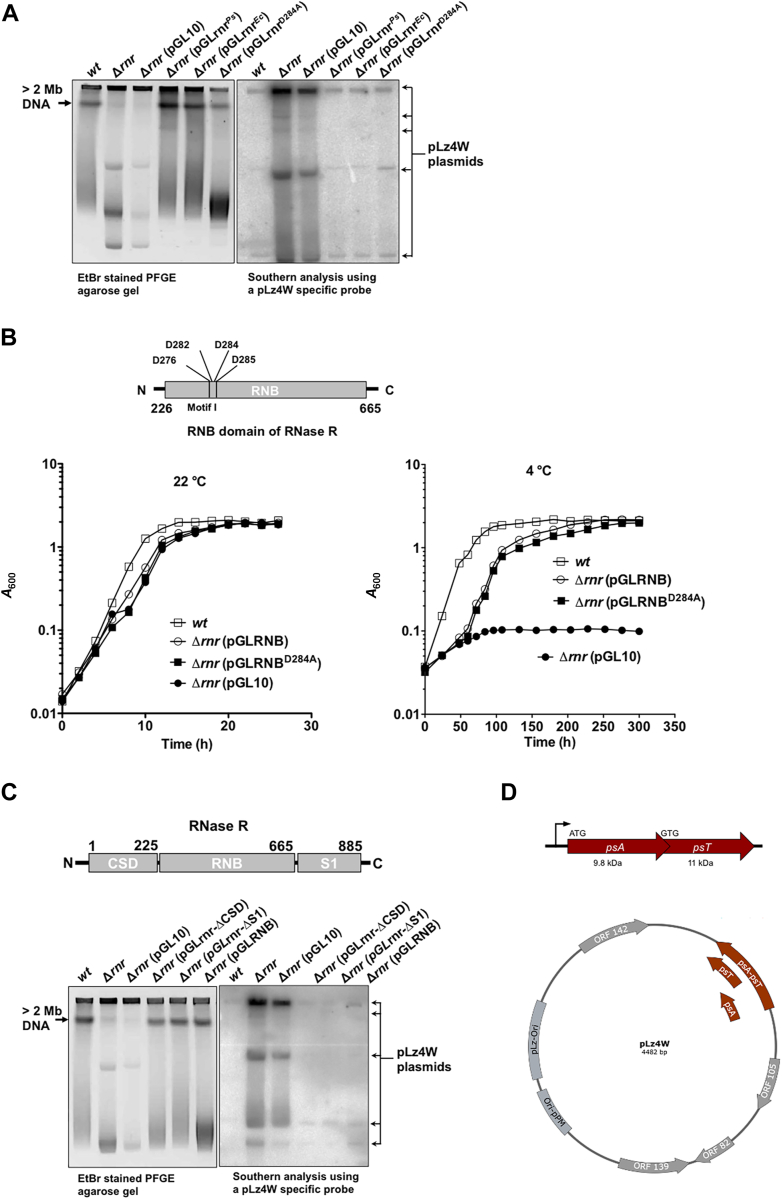
**Analysis of RNase R deleted *P. syringae* Lz4W strain complemented with RNase R variants.***A*, PFGE and Southern analysis of cellular DNAs of Δ*rnr* cells complemented with RNase R^Ps^, RNase R^Ec^, and RNase R^D284A^ mutants at 4 °C. Accumulation of discrete bands pLz4W DNA were observed only in Δ*rnr* and Δ*rnr* cells complemented with empty pGL10 vector. *B*, *top panel*, a schematic representation of the RNB domain of RNase R^Ps^, in which, the catalytically important aspartic acids of motif-1 of the RNB domain are shown. *Bottom panel*, growth profiles of *wt* and Δ*rnr* strains expressing RNB domain (pGLRNB) and the RNB domain with D284A mutation (pGLRNB^D284A^) at 22 °C and 4 °C. Growth was determined by measuring *A*_600_ at regular time intervals as indicated. *C*, *top panel*, a schematic representation of domain organization in RNase R^Ps^. The domains of RNase R^Ps^ along with amino acids residues’ number and the N and C termini are shown. *Bottom panel*, PFGE and Southern analysis of cellular DNAs of Δ*rnr* cells complemented with domain/s deleted mutants of RNase R^Ps^ at 4 °C. The CSD domain (Δ*CSD*), the S1 domain (Δ*S1*), and the CSD and S1 domains deleted (RNB) mutants of RNase R^Ps^ do not accumulate pLz4W-derived DNA fragments at 4 °C. *D*, a schematic representation of the indigenous pLz4W plasmid of *P. syringae* Lz4W. The *psA–psT* operon, open-reading frames, and other genetic elements are shown. Organization of the operon having *psA* and *psT* genes with overlapping reading frame under a common promoter is also depicted. The starting codons and predicted protein sizes of PsA antitoxin and PsT toxin are indicated. CSD, cold-shock domain; PFGE, pulsed-field gel electrophoresis; pLz4W, plasmid of *P. syringae* Lz4W; psA–psT, *P. syringae* antitoxin - *P. syringae* toxin system; RNase R^Ec^*, Escherichia coli* RNase R; RNase R^Ps^, RNase R of *P. syringae*.

To understand the role of RNase R in general and its functional implications in increased levels of pLz4W in *Δrnr* cells at low temperature, we expressed RNase R^Ps^, *Escherichia coli* RNase R (RNase R^Ec^), and the exoribonuclease defective mutant of RNase R^Ps^ (RNase R^D284A^) in the *Δrnr* strain of *P. syringae*. Our results show that the expression of both RNase R^Ps^ and RNase R^Ec^ alleviated the levels of pLz4W (Fig. 3*A*). Interestingly, the exoribonuclease-defective RNase R^D284A^ mutant also suppressed the high levels of pLz4W at 4 °C (Fig. 3*A*), indicating that the exoribonuclease activity of RNase R is not required for the maintenance of pLz4W at low temperature.

Note that the increased levels of linearized chromosomal DNA (>2 mb in size) observed in *wt* cells and the complemented *Δrnr* strains (Fig. 3*A*, left panel) are due to the low-temperature–induced replication fork collapse during active DNA replication as observed earlier (19). The *rnr* inactivated strains with/without the empty plasmid (control) did not show these chromosomal DNA fragments, possibly due to their inability to replicate at low temperature (Fig. 3*A*, left panel). Additionally, the DNA fragments of size ∼48.5 kb observed in the *Δrnr* (pGLrnr^D284A^) strain on the EtBr-stained PFGE agarose gel (Fig. 3*A*, left panel) are the broken chromosomal fragments similar to the fragments observed in *ΔrecCBD* cells grown at 4 °C (Fig. 2*A*) as discussed earlier (17, 19).

### The expression of the RNB domain of RNase R alone can also alleviate the levels of pLz4W plasmid

RNase R^Ps^ is a multidomain protein comprised of three domains: the N-terminal RNA-binding cold-shock domain (CSD), the central nuclease domain (RNB), and the C-terminal RNA binding S1 domain (S1) (27). A recent study showed that the expression of RNB domain alone (of RNase R^Ps^ or of RNase R^Ec^) rescues the cold sensitivity of *Δrnr P. syringae* cells (33). We further examined the ability of the RNB domain with exoribonuclease-defective mutation (RNB^D284A^) in rescuing the cold sensitivity of *Δrnr* cells. Our results show that the RNB domain with the D284A mutation also rescues the *Δrnr* mutant from cold sensitivity (Fig. 3*B*). This suggests that the RNB domain alone with no exoribonuclease function is sufficient to support the *P. syringae*’s growth at low temperature.

To understand the functional role of each domain in increased levels of pLz4W, we expressed RNase R without the CSD domain (pGLrnr-ΔCSD), RNase R without the S1 domain (pGLrnr-ΔS1) and RNase R with the RNB domain alone (without the CSD and S1 domains; pGLRNB) in *Δrnr P. syringae* cells. Our results show that the RNB domain alone, without the CSD and S1 domains, is sufficient to alleviate the increased levels pLz4W at low temperature in *Δrnr* cells (Fig. 3*C*). This further establishes a link between the maintenance of pLz4W levels and a putative role of the RNB domain without exoribonuclease function in *P. syringae’s* growth at low temperature.

### pLz4W, an indigenous plasmid of *P. syringae*, harbors a type II TA system

pLz4W is an indigenous pLz4W (9). To understand the reason for increased copy numbers of pLz4W in *Δrnr* cells at low temperature, we analyzed the genes encoded by pLz4W. Notably, pLz4W harbors a type II TA system related to the RelE/ParE family toxin (Fig. 3*D*). We designated this type II TA system as psA–psT (*P. syringae* antitoxin–*P. syringae* toxin system). The type II TA system is a small genetic element composed of a toxin protein and its cognate antitoxin protein (34). The pLz4W plasmid map with the *psA–psT* operon is shown in the Fig. 3*D*. The type II TA system consists of two genes (*psA* and *psT*) with overlapped reading frames (of 38 nucleotides) driven by a common promoter. The *psA* is 264 bp in size and encodes for PsA antitoxin of ribbon-helix-helix protein of the CopG family. The psT is 291 bp, which encodes for PsT protein of RelE/ParE family. The PsA and PsT proteins are estimated to be 9.8 and ∼11 kDa in size, respectively (Fig. 3*D*).

### pLz4W is a stable and low copy number plasmid

To determine the copy of number of pLz4W, the reads from the whole-genome sequence were aligned to the *P. syringae* Lz4W chromosomal sequence (NCBI Acc No. CP017432.1) and the pLz4W plasmid sequence (MF768471.1) using Bowtie 2 (35). The pLz4W plasmid length is 4482 bp; hence, a bed file was created with intervals of 4482 bp for the chromosome. The bed file was used to calculate the number of reads aligning to the chromosomal sequence with the interval length same as the plasmid length of 4482 bp using BEDTools (36). The average number of reads aligning at the 4482 bp intervals in the chromosome from the chromosomal alignment were 33,156, and the number of reads aligning to pLz4W from the plasmid alignment were 322,697. The ratio of plasmid reads to chromosomal reads were found to be 9.73, indicating the copy number of pLz4W in wildtype *P. syringae* cells is ∼10.

Since pLz4W appears to play a role in the cold sensitivity of *P. syringae* lacking RNase R, we also tried to cure the pLz4W plasmid from *P. syringae* cells using curing agents such as ethidium bromide and acridine orange. We failed to cure pLz4W from *P. syringae* cells. Since, the pLz4W plasmid harbors a type II TA system, the phenomenon of postsegregational killing may have played a role in prevention of its loss (37).

### Antitoxin expression suppresses the cold sensitivity and averts cell death of RNase R-deleted *P. syringae* cells at low temperature

The *rnr* mutant of *P. syringae* is highly cold sensitive, and 95% of cells die at 4 °C after 144 h of incubation (23). Although the reasons for cell death of *Δrnr* cells at low temperature are still unknown, our results now reveal that *Δrnr* cells accumulate high levels of various forms of pLz4W upon incubation at low temperature. Since the pLz4W plasmid harbors a type II TA system, we speculated that the *psA–psT* TA system could have a role in the cold-induced cell death of *Δrnr* cells. In type II TA systems, antitoxins are proposed to be less stable and susceptible for proteolytic degradation. The antitoxin degradation leads to the release of toxins to act on their cellular target, causing growth arrest or cell death (37, 38). This could also be a scenario for the *psA–psT* TA system in the *rnr* mutant of *P. syringae* at low temperature. Hence, we overexpressed PsA antitoxin on a low-copy expression vector pGL10 (pGLpsA) to circumvent the lethal effects of PsT toxin, if any.

**Figure 4:**
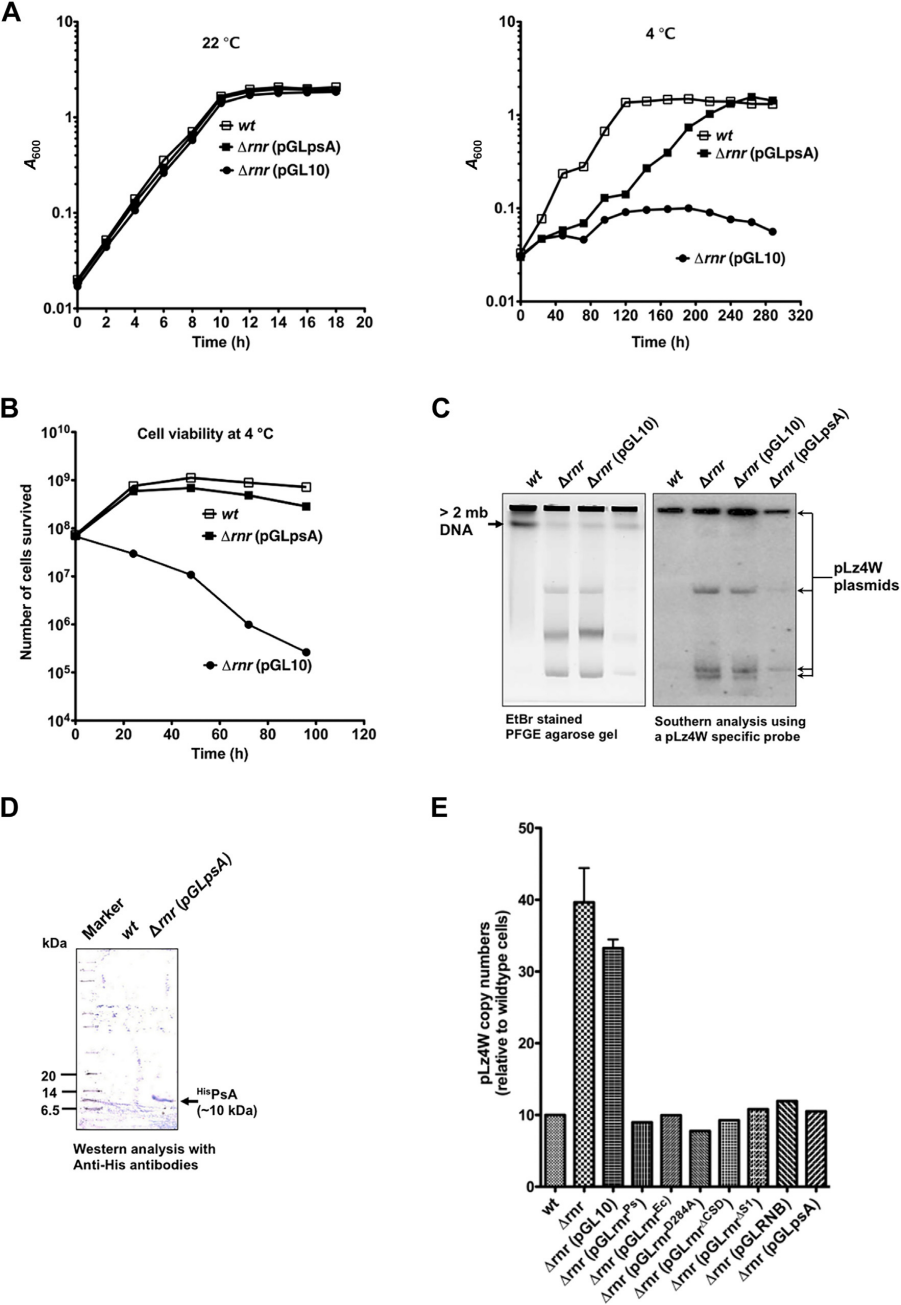
**PsA antitoxin expression alleviates the cold sensitivity of Δ*rnr* strain.***A*, growth profiles of *wt*, Δ*rnr* stain with an empty expression vector (pGL10), and Δ*rnr* strain expressing psA antitoxin (pGLpsA) determined at 22 °C and 4 °C. Growth was determined by *A*_600_ at regular time intervals as indicated. *B*, PsA antitoxin restores the cell viability of the Δ*rnr* strain similar to wildtype at 4 °C. Cells grown at 22 °C till *A*_600_ is 0.6, then were shifted to 4 °C and incubated for the indicated times. Aliquots of cells were collected at every 24 h, and the colony forming units (cfu/ml) were determined. *C*, PFGE and Southern analysis of cellular DNA of the Δ*rnr* strain expressing PsA antitoxin. PsA antitoxin expression reduces the copy number of pLz4W in the Δ*rnr* strain at 4 °C. *D*, Western analysis showing the expression of PsA antitoxin protein from the pGLpsA vector in Δ*rnr* cells. Expression of His-tag PsA antitoxin from the pGLpsA vector was determined by Western analysis using polyclonal antibodies specific to the 6x-His-tag. A protein marker with proteins of sizes 20, 14, and 6.5 are indicated. *E*, the copy number of pLz4W is reduced to the wildtype levels when RNase R and its derivatives are expressed *in trans*. Quantification of pLz4W from the PFGE-Southern hybridized gels was carried out using ImageJ2. For the *wt*, Δ*rnr*, and Δ*rnr*(pGL10) strains, the mean ± SD were calculated from the three independent experiments. The relative amount of pLz4W was plotted for the indicated strains compared to wildtype. PFGE, pulsed-field gel electrophoresis; pLz4W, plasmid of *P. syringae* Lz4W.

Interestingly, the overexpression of PsA antitoxin improved the growth of the *Δrnr* strain at low temperature (Fig. 4*A*, right panel). Importantly, the PsA overexpression restored the cell viability of *Δrnr* cells similar to that of the wildtype cells at 4 °C (Fig. 4*B*). In addition, *Δrnr* cells expressing PsA antitoxin showed reduced levels of pLz4W similar to wildtype cells at 4 °C (Fig. 4*C*). The additional DNA bands in the EtBr-stained PFGE agarose gel seen in the Fig. 4*C*-left panel, but not highlighted by the pLz4W signal in the Fig. 4*C*-right panel are the broken chromosomal fragments originated from the impaired DNA repair process in the *Δrnr* and *Δrnr* (pGL10) at 4 °C as mentioned above. The expression of PsA protein from the pGL10 expression vector was confirmed by Western analysis using anti-His antibodies (Fig. 4*D*) as described in Experimental procedures. These results imply that the increase in the copy number of pLz4W are linked to cell death of the *rnr* mutant of *P. syringae* at low temperature, possibly due to the activation of the type II *psA–psT* TA system and altered expression of toxin and antitoxin proteins.

### RNase R regulates the copy number of pLz4W and is critical for *P. syringae’s* growth at low temperature

*P. syringae* Lz4W contains ∼10 copies of pLz4W plasmid per cell. The southern hybridization of PFGE gels using a probe specific to pLz4W indicated that RNase R-deleted *P. syringae* (*Δrnr*) cells accumulate pLz4W plasmid at 4 °C (Figs. 2*C*, 3*C* and 4*C*). We analyzed these Southern blots specific to the pLz4W plasmid and quantified the levels of pLz4W relative to wildtype. As shown in Fig. 4*E*, data suggest that pLz4W copy number is increased by ∼4-fold in *Δrnr* cells compared to wildtype cells at 4 °C. The copies of pLz4W are increased from ∼10 copies to ∼40 copies/per cell in *Δrnr* cells at 4 °C (Fig. 4*E*). Whereas the copies of pLz4W were restored back to ∼10 copies/cell in *Δrnr* cells, when RNase R^Ps^, RNase R^Ec^, RNase R^D284A^, RNB domain of RNase R^Ps^, RNB^D284A^, and psT antitoxin proteins are expressed *in trans* (Fig. 4*E*). This indicate that RNase R and its derivatives regulate the copy number of pLz4W. This further confirms a previously unknown link between the regulation of copy number of pLz4W harboring the type II psA–psT system and the exoribonuclease RNase R.

## Discussion

Bacteria living in extreme environmental conditions often need structural and functional adaptations to overcome the challenges posed under these conditions. The maintenance of DNA topology, DNA replication, RNA metabolism, and protein synthesis are crucial under these stressful environmental conditions. A faithful completion of DNA replication and maintenance of quality control of RNA metabolism have been found to be essential for the survival of Antarctic psychrotolerant *P. syringae* Lz4W at low temperature (2).

In bacteria, the exoribonuclease RNase R is important to cope with multiple stress conditions (27, 39). It is known to be upregulated in response to starvation, cold shock, and during stationary phase growth (39, 40, 41). Notably, the RNase R^Ps^ is not upregulated by cold shock, stationary phase growth, oxidative stress, and DNA damage response (23, 27). However, RNase R^Ps^ is essential for the growth of *P. syringae* at low temperature (23). The inactivation of the *rnr* gene encoding RNase R leads to cold sensitivity, and *Δrnr* cells die upon incubation at low temperature (23). RNase R also protects *P. syringae* cells from DNA damage and oxidative stress (27). To understand the function of RNase R^Ps^ in genome maintenance, we resolved the entire cellular DNA of *Δrnr* cells grown at 22 °C and at 4 °C by PFGE. Surprisingly, we observed that *Δrnr* cells accumulate DNA specific to the indigenous pLz4W plasmid at low temperature.

In *Pseudoalteromonas rubra*, the type II PrpT-prpA TA system on a plasmid directly controls plasmid replication. The deletion of PrpA antitoxin, a transcription repressor, leads to increase in the copy number of the plasmid (42). PrpT toxin of *P. rubra* resembles ParE toxin that inhibits gyrase’s function, and PrpA antitoxin belongs to the CopG family transcription regulators (42, 43). In the plasmid-borne psA–psT TA system of *P. syringae*, PsA toxin resembles ParE toxin, and PsT antitoxin is a CopG-like transcriptional repressor. Although the mechanism by which the increase in copy number of pLz4W occurs in the absence of RNase R in *P. syringae* at low temperature is not clearly known; we speculate that the psA–psT system of *P. syringae* on pLz4W controls its replication to maintain the copy number similar to the type II PrpT-prpA system on the plasmid of *P. rubra* (42).

In type II TA system, the activity of stable toxin is generally inhibited by binding with less-stable antitoxin to form a neutral complex (44). In response to specific cellular and environmental cues, increased degradation of less stable antitoxin by proteolytic degradation enables stable toxin to exert lethal effects on cells causing either growth arrest or cell death (38). Antitoxins also act as transcription repressors and autoregulate TA function at transcriptional level (34). TA systems also work as stress response modules (34). Hence, a decrease in antitoxin level, by any means, is detrimental to cells. This could also be the scenario in the case of RNase R-deleted *P. syringae* cells at low temperature, where the increased copies of pLz4W containing psA–psT TAs is correlated with their cell death at low temperature. Our results show that the ectopic expression of PsA antitoxin from a plasmid alleviates the cold sensitivity and restores the cell viability of *Δrnr cells* comparable to *wt* cells at low temperature. The ability of *Δrnr P. syringae* cells to grow at low temperature and regaining the cell viability when PsA antitoxin is overexpressed confirms that the psA–psT TA system is indeed responsible for the loss of viability of *Δrnr* cells at low temperature.

In *P. syringae*, RNase R is a processive, hydrolytic, 3′ – 5′ exoribonuclease associates with a novel degradosome complex and plays an important role in RNA metabolism (25). It is a sequence nonspecific exoribonuclease and degrades through the secondary structures of dsRNA (26). RNase R also plays a novel role in the maturation of 16S and 5S ribosomal RNA. The unprocessed 16S RNAs accumulate in polysomes affecting protein synthesis at low temperature in *rnr* mutant *P. syringae* cells (23). *In E. coli*, Hfq and RNase R have also been shown to be involved in the maturation of 16S and 23S rRNA precursors and ribosome assembly (45). In *Mycoplasma genitalium*, RNase R is the only exoribonuclease present and is required for cell survival (46). Since the exoribonuclease function of RNase R is imperative in RNA metabolism, we investigated the role of exoribonuclease activity of RNase R^Ps^ in the cold adaptation of *P. syringae*. Here, we show that *Δrnr* cells expressing exoribonuclease-defective RNase R^D284A^ and the RNB domain alone with D284A mutation grow proficiently both at 22 °C and 4 °C. This indicates that the exoribonuclease function of RNase R is not required for *P. syringae’s* growth at low temperature, despite it is being imperative for processing, maturation of ribosomal RNAs, and assembly of ribosomes.

In *E. coli*, RNase R is shown to possess both the helicase and exoribonuclease activities. The helicase function of RNase R is independent of its exoribonuclease activity, and it is shown to be important for complementing the cold shock function of CsdA, a RNA helicase (16, 24). RNase R^Ec^ with mutations in its ATP-binding Walker motifs exhibited growth defects at low temperatures (24). Further, the structural and biochemical analyses of RNase R^Ec^ have revealed that the RNase R appears to have a tri-helix ‘wedge’ region in the RNB domain and is shown to be important for RNA unwinding (47). Recently, a similar tri-helix ‘wedge’ region in the RNB domain of RNase R^Ps^ is also predicted and proposed to have a putative role in RNA unwinding (27). Notably, the RNase R^Ec^ and the RNB domains of RNase R^Ec^ and RNase R^Ps^ have been recently shown to rescue the cold sensitivity of *Δrnr* cells of *P. syringae* (33). Low temperatures can stabilize the secondary structures of RNA, rendering the RNA unwinding function more vital for the survival of living organisms at low temperatures. Although we have not evaluated the importance of the helicase function of RNase R^Ps^ directly, the ability of RNase R^Ec^ and the RNB domain of RNase R^Ec^ to complement the cold sensitivity of *Δrnr* cells of *P. syringae* indicates that the RNA unwinding function of RNase R is likely to be important for the survival of *P. syringae* Lz4W at low temperature.

In this study, we have uncovered a previously unknown physiological/functional relationship between the RNase R–associated cold sensitivity and the plasmid-borne psA–psT, a type II TA system in *P. syringae* Lz4W. Our study has also revealed that the exoribonuclease function of RNase R and its role in processing and maturation of 16S RNA is not essential for the survival of *P. syringae* Lz4W at low temperature.

## Experimental procedures

### Bacterial strains, plasmids, and growth conditions

Bacterial strains, plasmids, and oligonucleotides used in this study are listed in Table 1. *P. syringae* Lz4W cells were grown in Antarctic bacterial medium (5g liter^−1^ peptone and 2g liter^−1^ yeast extract) at both 22 °C and 4 °C. *E. coli* strains were grown in LB medium at 37 °C. For the complementation studies, plasmids were transferred into *P. syringae* Lz4W from IncQ mobilizing strain *E. coli* S17-1. The growth media were supplemented with the following antibiotics as needed: 100 μg ml^−1^ ampicillin, 50 μg ml^−1^ kanamycin, and 20 μg ml^−1^ tetracycline. For the growth analysis, cultures were grown overnight in AB media and were inoculated into a fresh AB media at 1:100 dilutions, and the *A*_600_ was measured at the time intervals as indicated.

**Table 1:** Bacterial strains, plasmids, and oligonucleotides used in the study

Bacterial strains		
Bacterial strain	Description	Source/reference
*P. syringae* Lz4W	Wild type, Antarctic isolate	(1)
*Δrnr*	*P. syringae* Lz4W but *Δrnr::tet*^*r*^	(23)
S17-1	*F*^*−*^*pro recA1 (r*^*−*^*m*^*−*^*) RP4-2* integrated *(Tc::Mu) (Km::Tn7) [Smr Tpr] E. coli* strain: used as plasmid mobilizing strain	(43)

Plasmids		
Plasmid	Description	Source
pGL10	Broad-host-range vector *with IncP* replicon	(44)
pGL*rnr*	pGL10 vector expressing His-tagged RNase R of *P. syringae* Lz4W	(25)
pGL*rnr*^Ec^	pGL10 vector expressing His-tagged RNase R of *E. coli*	(27)
pGLrnr-*ΔCSD*	pGL10 vector expressing His-tagged RNase R without CSD domain	(27)
pGLrnr-*ΔS1*	pGL10 vector expressing His-tagged RNase R without S1 domain	(27)
pGL*RNB*	pGL10 vector expressing His-tagged RNB domain of RNase R without CSD and S1 domains	(27)
pGL*rnr*^D284A^	pGL10 vector expressing His-tagged RNase R with D284A mutation	(27)
pGL*psA*	pGL10 vector expressing His-tagged psA antitoxin	This study
pET*psA*^*His*^	pET28a vector expressing N-terminal His-tagged psA antitoxin	This study
pLz4W	An indigenous *P. syringae* Lz4W plasmid (4482 bp in size) harboring the type II *psA–psT* system	(ncbi.nlm.nih.gov/nuccore/MF768471.1).

Oligonucleotides and primers		
Oligonucleotides/primers	Sequence (5′ → 3′)	Description
PsA_FP_NI	CGTATACATATGGCCTCGCCTGTCCTGTCTTTTCG	Forward primer for amplifying the *PsA* gene with *NdeI* site
PasA_RP_ScI	CATGAGAGCTCTCACTGTCCGCTTTGCTGGTTAATGC	Reverse primer for amplifying the *PsA* gene with *SacI* site
Probe-P1	GAGAAAACCCTAAACTTGGCTCAGCAA	A probe to identify the unprocessed region of 16S RNA by Northern analysis
Probe-P2	CCTGCCGCCAGCGTTCAATCTGAGCCA	A probe to identify the processed region of 16S RNA by Northern analysis

### Reagents and general DNA recombinant methods

The molecular biology techniques such as genomic DNA isolation, restriction enzyme digestion, PCR, RNA isolation, Southern and Northern hybridization, Western analysis, DNA ligation, transformation, and conjugation were performed as described earlier (48). All restriction enzymes, T4 DNA ligase, and T4 PNK were purchased from New England Biolabs. Oligos used in the study were purchased from Bioserve India. PCR was performed using proof-reading Pfx DNA polymerase from Invitrogen. The ABI Prism dye terminator cycle sequencing method (PerkinElmer) was used for DNA sequencing, and automated DNA sequencer (ABI model 3700; Applied Biosystems) was used for the analysis. Antibodies were from Santa Cruz Biotechnology.

The *psA* gene was PCR amplified using the primers PsA_FP_NI and PsA_RP_ScI (Table 1) using the pLz4W plasmid isolated from wildtype *P. syringae* Lz4W cells. The amplified *pasA* gene was cloned in NdeI and SacI sites of pET28b expression vector. The expression of N-terminally His-tagged PsA protein was examined by Western analysis using anti-His antibodies as described earlier (27). For expressing psA antitoxin in the *Δrnr* strain, the *psA* gene was subcloned into the XbaI and SacI sites of pGL10, and the resultant pGL*psA* plasmid was mobilized into the Δ*rnr* strain by conjugation as described earlier (49).

### Growth analysis of bacterial cultures by measuring the absorbance

For the growth analysis, *wt*, Δ*rnr*, Δ*rnr*(pGL10), Δ*rnr*(pGL*psA*), and Δ*rnr*(pGL*rnr*^*D284A*^) cells of *P. syringae* Lz4W were grown at 22 °C overnight. 1% of the overnight grown primary cultures were inoculated into a fresh AB media and incubated at 22 °C and 4 °C with proper aeration. The absorbance at 600 nm (*A*_600_) of cells was measured at specified time-intervals. The culture media was supplemented with ampicillin 100 μg/ml and kanamycin 50 μg/ml as needed. The graphs were plotted using GraphPad Prism v.10.0.0.

### PFGE and Southern hybridization

For the PFGE analysis, cells were grown overnight in AB media containing appropriate antibiotics. The cultures were reinoculated at 1:100 dilution in a fresh AB media. Cells were harvested at an exponential phase (*A*_600_ = ∼0.6) and embedded in 1% w/v LGT agarose blocks (Lonza). Electrophoresis was carried out in CHEF-DRII (Bio-Rad) at a constant voltage of 180 V, with increasing the pulse time of 60 to 120 s over a period of 20 h at 14 °C. Whenever mentioned, the DNA in PFGE gels was transferred onto the N+ membrane in the presence of an alkaline transfer buffer containing 0.4M NaOH and 1M NaCl. The probes (Table 1) were labeled with [α-^32^P]-ATP using a random primer labeling kit from JONAKI, BARC. For generating probes specific to the genomic and pLz4W plasmid DNA, a full-length *recA* gene and the entire pLz4W plasmid DNA were used as templates. The hybridization reaction was carried out at 65 °C for 16 to 18 h in 0.5M sodium phosphate buffer containing 7% w/v SDS and 1 mM EDTA. The membranes were washed thrice at 65 °C for 15 min with 40 mM sodium phosphate buffer containing 1% w/v SDS and 1 mM EDTA. Radioactive signals were detected by phosphorimager (Fuji FLA 3000). Quantification of Southern blots was done using the ImageJ2 version: 2.14.0/1.54f software.

### RNA isolation and Northern analysis

RNA was isolated using the hot-phenol method as described earlier (23). Briefly, the cells were grown until *A*_600_ = 0.6. RNA was isolated from 3 ml culture by treating with equal volume of acid-phenol (pH 5.5). The aqueous phase was precipitated using absolute ethanol. The RNA pellet was washed with 70% ethanol, dried, and finally resuspended in 50 μl RNase-free autoclaved Milli-Q water.

The Northern hybridization analysis was carried as described earlier (23). Briefly, wildtype and the mutant strains of *P. syringae* Lz4W were grown at 22 °C until *A*_600_ is ∼0.6. The cultures were then shifted to 4 °C. At every 24 h, 6 ml culture was removed and, of which, 3 ml was centrifuged immediately, and the remaining 3 ml culture was treated with 400 ug/ml of rifampicin at 4 °C for 60 min. Total RNA was isolated from both samples, resolved on 1% w/v agarose gel in Tris-borate-EDTA buffer, and transferred on to a Hybond N^+^ membrane. The hybridization was carried out using 5′-end [γ- ^32^P]-labeled oligonucleotide probes (Table 1).

### Cell viability test

The cell viability of *wt*, Δ*rnr*, Δ*rnr*(pGL10), and Δ*rnr*(pGL*psA*) cells of *P. syringae* Lz4W was calculated by measuring the colony-forming units (cfu/ml) at low temperature. Cells were grown overnight at 22 °C with appropriate antibiotics. The overnight grown cultures were diluted 1:100 into a fresh AB media containing appropriate antibiotics and incubated at 22 °C with proper aeration until they reached *A*_600_ of ∼0.6. Further, cells were shifted to 4 °C incubation. Samples were collected every 24 h, and appropriate dilutions were made and plated on Antarctic bacterial medium agar plates. The plates were incubated at 22 °C for 48 h. The percentage viability of each strain was calculated by considering their respective cfu before shifting to 4 °C as 100%.

### Statistical analysis

Data were calculated and analyzed by using Prism 10 (GraphPad Software Inc).

## Data availability

All the data generated and analyzed in the study are included in this published article.

## Supporting information

This article contains supporting information.

## Conflict of interest

The authors have no conflict of interest with the contents of this article.
