# Supplementary material for: A type II toxin–antitoxin system is responsible for the cell death at low temperature in Pseudomonas syringae Lz4W lacking RNase R
Source: J Biol Chem. 2024 Jul 25;300(8):107600. doi: 10.1016/j.jbc.2024.107600 (PMC11375266; doi:10.1016/j.jbc.2024.107600)
Supplement: Supplemental Figure S1 [file mmc1.pdf]

Supporting information for

A type II toxin-antitoxin system is responsible for the cell death at low temperature in *Pseudomonas syringae* Lz4W lacking RNase R.

Pragya Mittal[1, 2*], Anurag K Sinha[1, 3], Apuratha Pandiyan[1, 4], Leela Kumari[1], Malay K Ray[1], and Theetha L Pavankumar[1,5*]

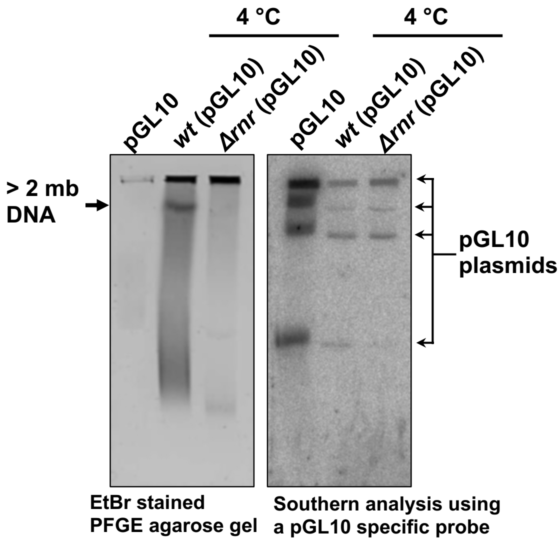

Figure S1. PFGE and Southern analysis of cellular DNAs of *wt* and *Δ*rnr cells harboring a broad-host range pGL10 vector grown at 4 °C . A purified pGL10 plasmid of 8.5 kb in size was run under the PFGE conditions as a control (lane - pGL10). Left panel is the ethidium bromide stained PFGE gel and the right panel is Southern analysis of the PFGE gel using a probe specific to pGL10 plasmid.

Figure S1
